# Ablation of Ventricular Arrhythmias in GUCH: The Surgical Scar and the Second Substrate

**DOI:** 10.1016/s0972-6292(16)30457-0

**Published:** 2012-01-31

**Authors:** Narayanan Namboodiri

**Affiliations:** Sree Chitra Tirunal Institute for Medical Sciences and Technology, Trivandrum, Kerala, India

**Keywords:** ventricular tachycardia, catheter ablation

Patients with grown-up congenital heart (GUCH) disease pose a few unique problems for the cardiac electrophysiologist due to the myocardial substrate with high propensity for arrhythmias. Arrhythmias are the main reason for hospital admission in these patients and, unfortunately, in a significant proportion of these patients, these arrhythmic events could be life-threatening [1]. Furthermore, the onset of arrhythmias may herald the hemodynamic decompensation in a sizable segment of the population with already compromised functional status. The abnormal circulation, in turn, may amplify the hemodynamic effect of arrhythmias in these patients with unrepaired or partially repaired abnormal cardiac anatomy.

Previous studies into the natural history of patients with GUCH have been helpful in identifying the likely risk factors for arrhythmogenesis, and the associated risk of sudden cardiac death (SCD) in them [2-4]. Clinical variables like older age at repair, ventricular dysfunction, and need of ventriculotomy at repair are known to adversely affect the survival in patients with GUCH [2-4]. In majority, the increased arrhythmogenecity, at cellular level, is mediated by the co-existing myocardial fibrosis. Ventricular fibrosis suggested by cardiovascular magnetic resonance is known to be an important marker of poor survival in adults with repaired tetralogy of Fallot (TOF) [4]. Additional evidence for myocardial fibrosis in these patients is that re-entrant mechanisms, as often seen with scarring, account for majority of their arrhythmias.

As myocardial scarring can be taken as a surrogate marker of arrhythmogenesis in these patients, it is important to recognise the factors contributing to its development in them. The patients who had undergone ventriculotomy and / or patching for ventricular septal defects, who carry the highest risk of developing ventricular arrhythmias in GUCH, have a well-defined surgical scar. As described earlier for intraatrial-reentrant tachyarrhythmias associated with atriotomy scars, the macro-reentrant circuits in post-ventriculotomy patients tend to use narrow isthmuses within the surgical scars or between the scars and anatomical barriers like valves or patches for its perpetuation [6]. In either of the above mechanisms, the surgical scars tend to determine the circuits and thereby, the targets of ablation. Zeppenfeld et al had demonstrated that the placement of a single ventriculotomy, outflow tract patch, or transannular patch, along with closure of the ventricular septal defect, would be expected to create only a limited number of possible tachycardia circuits [7]. However, diffuse or localised myocardial fibrosis- the second substrate apart from scar - can also contribute to arrhythmogenesis in a significant proportion of patients with GUCH. The abnormal hypertrophic response, chamber dilatation, myocardial disarray etc. could account for these additional factors in these patients with high likelihood of developing pressure and/or volume overload of cardiac chambers. Myocardial scarring secondary to hemodynamic factors have already been described in patients with GUCH with various underlying cardiac anomalies [8]. These include patients with corrected transposition, Ebstein's anomaly, single ventricle, Eissenmenger's syndrome and unrepaired TOF.

In this issue of the journal, Selvaraj et al describe a case where the circuit of the ventricular tachycardia was mapped to a site far away from scar in a patient who had undergone ventriculotomy as a part of surgical correction of double-chambered right ventricle [9]. Myocardial fibrosis, as defined by the voltage map settings, showed scar at right ventricular apex in this case. Due to the hemodynamic instability associated with the tachycardia, the authors could not perform detailed entrainment mapping techniques to delineate the circuits, and had to ablate based on pace mapping. Despite these limitations, the response to ablation at right ventricular apex was validated by the clinical response with no recurrence on follow up. The authors hypothesise the prolonged exposure of right ventricular apex to high pressures could account for the localised scarring noted in this case. This proposed association between this regional myocardial scarring unrelated to the prior ventriculotomy and the potential targets of successful ablation is important in an era where the hunt for the optimal targets of ablation in complex arrhythmias is continuing. This is more relevant in patients with GUCH and arrhythmia, where alternative therapeutic options are limited. The negative inotropic effects and safety concerns of antiarrhythmic drugs limit their wide usage, especially at high doses often required to prevent recurrence of arrhythmias in them. The indications for implantable defibrillators are also not well established. Due to these concerns, though the results of catheter-based ablation are far from satisfactory, electrophysiological testing and/or ablation are considered as a worthy option for symptomatic patients with suspected or documented ventricular arrhythmia. Identification of these additional mechanisms of arrhythmogenesis and possible targets of ablation carries greater significance in this scenario.

The therapeutic algorithms for the management of arrhythmias tend to differ in these patients owing to the heterogeneity of the anatomical and physiological substrate. Despite this limitation, this report underscores an important fact that the arrhythmogenic substrate in congenital heart disease may not necessarily be the ventriculotomy scar alone, and the second substrate -the fibrosis related to the myocardial remodelling due to chamber hypertrophy or dilatation - could be contributory in many. Duration of exposure of heart to the abnormal hemodynamic burden may be the most important factor determining the degree of remodelling and, thereby, fibrosis. The higher risk of SCD in TOF patients repaired after 20 years of age have already been described [3]. In a large, multicenter study, nearly 100% of patients with TOF who had their surgical procedure performed after 10 years of age had ventricular arrhythmias [10]. Similarly, electrical remodelling in atria is known to occur by adolescence in patients with atrial septal defects, and this has been found as a major risk factor for atrial arrhythmias in postoperative patients with delayed age at repair [11]. These facts clearly point towards the complementary role of myocardial fibrosis as an additional arrhythmogenic factor apart from surgical scar in these patients. However, more studies are required to identify the mechanisms of complex electrophysiological interaction between the scar and this 'second substrate' in initiation and perpetuation of these re-entrant arrhythmias in patients with congenital heart disease.

## References

[R1] Somerville J (1997). Management of adults with congenital heart disease: an increasing problem. Annu Rev Med.

[R2] Ghai A (2002). Left ventricular dysfunction is a risk factor for sudden cardiac death in adults late after repair of tetralogy of Fallot. J Am Coll Cardiol.

[R3] Gatzoulis MA (2000). Risk factors for arrhythmia and sudden cardiac death late after repair of tetralogy of Fallot: a multicentre study. Lancet.

[R4] Cullen S (1994). Prognostic significance of ventricular arrhythmia after repair of tetralogy of Fallot: a 12-year prospective study. J Am Coll Cardiol.

[R5] Babu-Narayan SV (2006). Ventricular fibrosis suggested by cardiovascular magnetic resonance in adults with repaired tetralogy of Fallot and its relationship to adverse markers of clinical outcome. Circulation.

[R6] Nakagawa H (2001). Characterization of reentrant circuit in macroreentrant right atrial tachycardia after surgical repair of congenital heart disease: isolated channels between scars allow "focal" ablation. Circulation.

[R7] Zeppenfeld K (2007). Catheter ablation of ventricular tachycardia after repair of congenital heart disease: electroanatomic identification of the critical right ventricular isthmus. Circulation.

[R8] Oechslin EN (2000). Mode of death in adults with congenital heart disease. Am J Cardiol.

[R9] Selvaraj RJ (2012). Ventricular tachycardia in repaired double chambered right ventricle - identification of the substrate and successful ablation. Indian Pacing Electrophysiol. J.

[R10] Chandar JS (1990). Ventricular arrhythmias in postoperative tetralogy of Fallot. Am J Cardiol.

[R11] Attie F (2001). Surgical treatment for secundum atrial septal defects in patients > 40 years old. A randomized clinical trial. J Am Coll Cardiol.

